# Supplementary material for: A Novel Wall-Associated Kinase TaWAK-5D600 Positively Participates in Defense against Sharp Eyespot and Fusarium Crown Rot in Wheat
Source: Int J Mol Sci. 2023 Mar 6;24(5):5060. doi: 10.3390/ijms24055060 (PMC10003040; doi:10.3390/ijms24055060)
Supplement: Supplementary file 1 [file ijms-24-05060-s001.zip › ijms-2243504-supplementary.pdf]

Supplementary material: Fig. S1 The infection types of Sharp eyespot in BSMV: GFP and BSMV: TaWAK-5D600 wheat seedlings.

Fig. S2 The infection types of *Fusarium* Crown Rot in BSMV: GFP or BSMV: TaWAK-5D600 wheat plants.

Fig. S3 Variation of *TaWAK5D600* in natural population.

Fig. S4 Fragment used for BSMV: TaWAK-5D600 and off-target prediction by si-Fi software.

Table S1. Ka, Ks, and Ka/Ks values of all 140 TaWAK proteins.

Table S2. The pairwise protein sequence comparison between the TaWAK-5D600 and other diseases resistant WAK kinase proteins in crop plants.

Table S3. Primers used in this study.

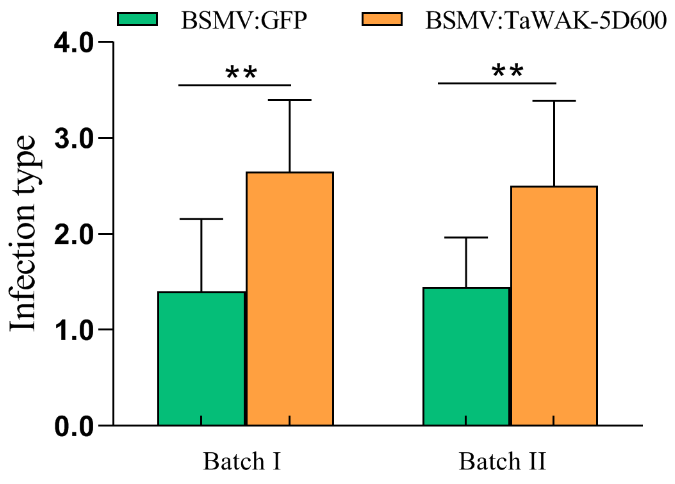

Figure S1. The infection types of sharp eyespot in BSMV: GFP and BSMV: TaWAK-5D600 wheat seedlings.

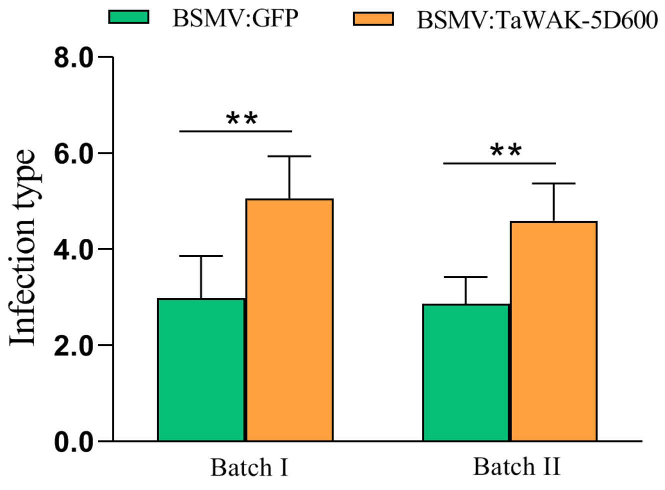

Figure S2. The infection types of *Fusarium* Crown Rot in BSMV: GFP or BSMV: TaWAK-5D600 wheat plants.

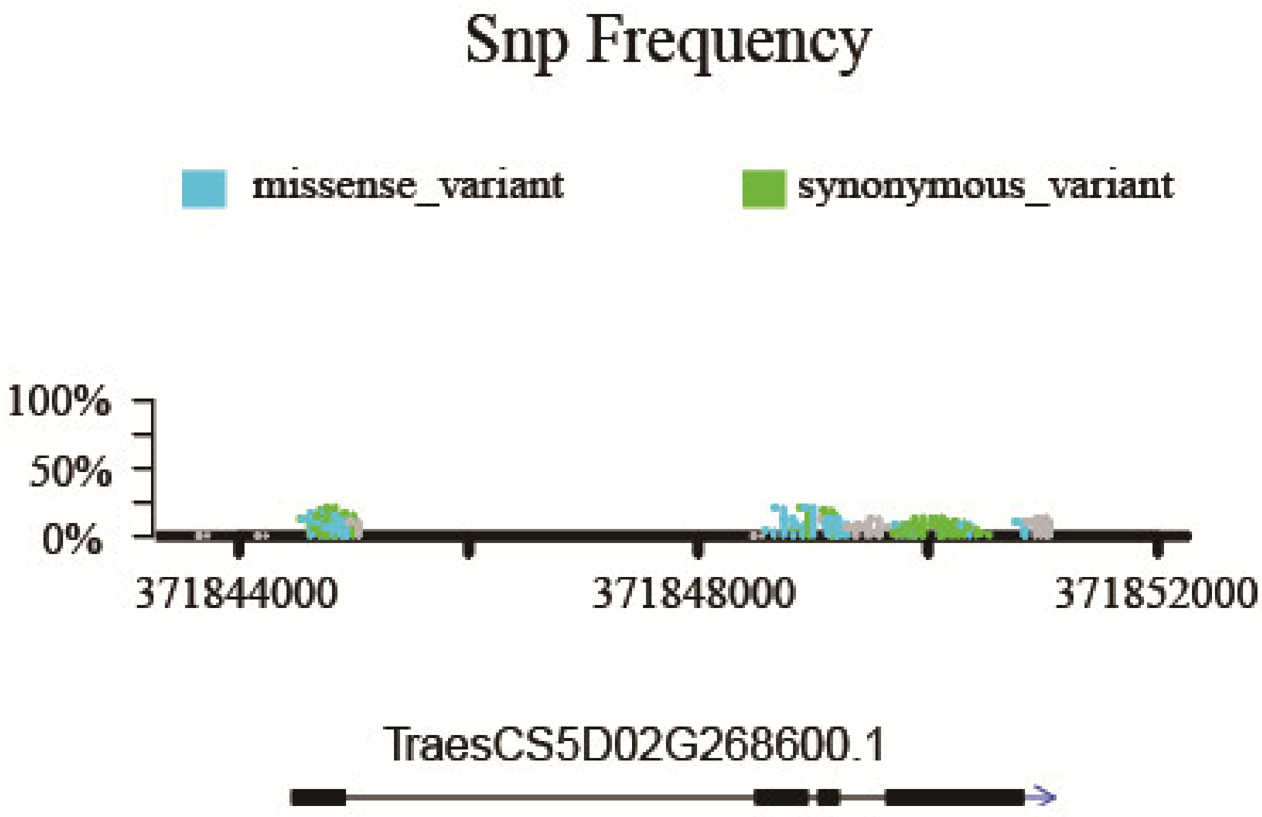

Figure S3. Variation of *TaWAK5D600* in natural population (http://wheat.cau.edu.cn/WheatUnion).

>VIGS-TaWAK-5D fragment (192 bp)

CATCCCGTTCCCGTACGGCGTCGGCAAGGAGTGCGCGTTACACGACGGCTTTAACCTCAACTGC
ACCACCGTGGAAGGCGTCGAGATGCCTCTTGCGGGGCATATCCAGGTGATTAAGATCTCTTTGGC
TAACTCCACAGCCACGTTGAATACCACGGCGATCTCGTGGAAGTGCTATCACCCAGCCACTGG

Targets	Total siRNA hits	Efficient siRNA hits
TraesCS5D02G268600.1	172	81
TraesCS5D02G256700.1	2	0
TraesCS5A02G249400.1	2	0
TraesCS5D02G256800.1	1	0

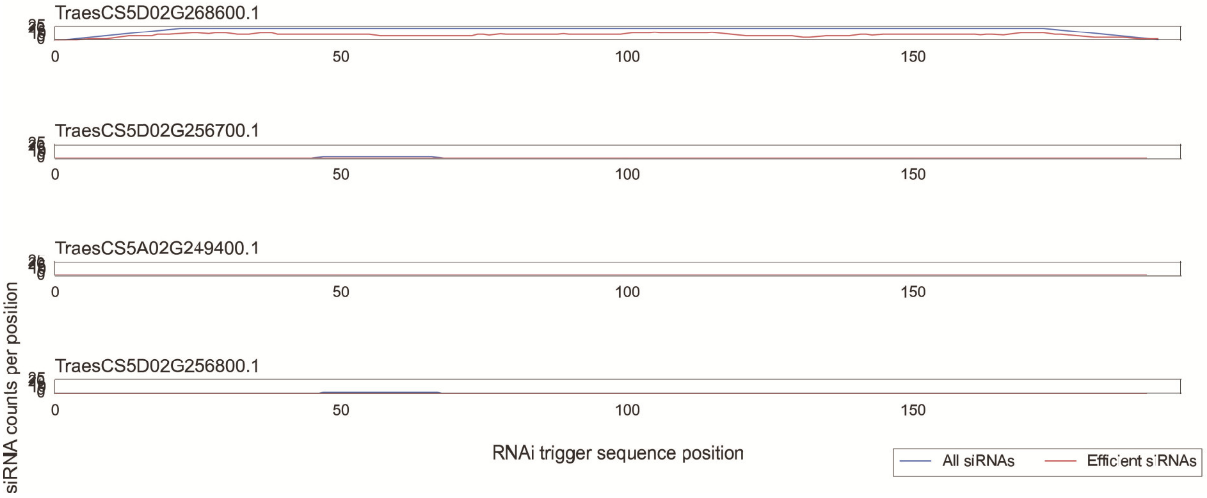

Figure S4. Fragment used for BSMV: TaWAK-5D600 and off-target prediction by si-Fi software.

Table S1. Ka, Ks, and Ka/Ks values of all 140 *TaWAK* genes

Gene ID	Molecular weight (kDa)	Isoelectric point (pI)	Amino acid sequences length (aa)
TraesCS2B02G059400.1	81.05	7.77	753
TraesCS2D02G046500.1	81.23	6.89	753
TraesCS6B02G073000.1	81.45	7.07	759
TraesCS6A02G054500.1	81.08	7.27	757
TraesCS6D02G056600.1	81.50	7.22	759
TraesCS2A02G047800.1	81.24	8.12	747
TraesCS2D02G045900.1	81.09	7.98	747
TraesCS5A02G365300.1	82.03	6.5	761
TraesCS5D02G373700.1	82.04	7.17	762
TraesCS5B02G366900.1	82.18	6.74	761
TraesCS6B02G059800.1	80.94	6.12	748
TraesCS6A02G043900.1	81.74	6.39	758
TraesCS6D02G050900.1	81.93	6.08	759
TraesCS6D02G046818.1	87.05	8.38	791
TraesCS1A02G340300.1	83.47	7.12	769
TraesCS1D02G342300.1	83.65	7.08	770
TraesCS1B02G352600.1	83.62	7.62	771
TraesCS7D02G463600.1	84.16	5.45	769
TraesCS5B02G367600.1	83.49	6.63	761
TraesCS7A02G242800.1	80.87	6.1	744
TraesCS7B02G148800.1	81.38	6.09	747
TraesCS7A02G242900.1	82.97	5.84	752
TraesCS7B02G148700.1	82.78	6.12	751
TraesCS5D02G330900.1	83.47	6.61	750
TraesCS5A02G323900.1	84.28	6.2	761
TraesCS5B02G324500.1	84.16	6.7	760
TraesCS5B02G324400.1	78.70	6.63	714
TraesCS5A02G323800.1	78.88	6.9	716
TraesCS5D02G330600.1	79.29	6.94	717
TraesCS2B02G464000.1	80.33	6.72	739
TraesCS2A02G442900.1	81.08	7.36	742
TraesCS2D02G442000.1	80.94	7.35	742
TraesCS6B02G040700.1	81.63	5.26	737
TraesCS6D02G032300.1	81.42	5.18	737
TraesCS3A02G006900.1	82.51	4.89	759
TraesCS3D02G003900.1	82.80	5.92	760

TraesCS5A02G261200.1	82.34	6.41	743
TraesCS5B02G259500.1	81.84	6.62	739
TraesCS5D02G268600.1	82.02	6.15	743
TraesCS5A02G249600.1	81.63	7.54	736
TraesCS5D02G256800.1	81.51	7.79	736
TraesCS5A02G249300.1	83.92	4.89	760
TraesCS5B02G247400.1	81.89	5.52	744
TraesCS5D02G256700.1	81.29	5.74	737
TraesCS6B02G256300.1	84.16	5.61	757
TraesCS6A02G225300.1	84.02	5.61	757
TraesCS6D02G210100.1	84.17	5.89	757
TraesCS6A02G075500.1	82.23	6.28	739
TraesCS6B02G101400.1	81.57	5.77	736
TraesCS6A02G027400.1	81.58	6.87	732
TraesCS1D02G037200.1	82.10	6.09	745
TraesCS7D02G136700.1	82.21	6.18	745
TraesCS1A02G035700.1	82.16	6.14	745
TraesCS1B02G009600.1	83.33	6.61	756
TraesCS2D02G377900.1	82.37	6.39	742
TraesCS2A02G381500.1	82.10	5.17	743
TraesCS2D02G378000.1	79.69	6.32	718
TraesCS2B02G489700.1	82.28	6.56	743
TraesCS2B02G580400.1	82.48	7.06	744
TraesCS2D02G550900.1	82.28	6.82	744
TraesCS5A02G274100.1	76.88	7.31	714
TraesCS5D02G281200.1	76.81	7.01	713
TraesCS6A02G376500.1	85.01	7.23	764
TraesCS6B02G414200.1	85.06	6.54	765
TraesCS6D02G361700.1	84.85	6.58	763
TraesCS6B02G413700.1	81.60	6.71	737
TraesCS6D02G361000.1	84.19	6.85	759
TraesCS6A02G376005.1	83.84	6.07	759
TraesCS6A02G376000.1	82.59	7.88	749
TraesCS6D02G361100.1	81.80	8.02	742
TraesCS6A02G376200.1	82.22	6.1	754
TraesCS6D02G361400.1	82.21	6.1	754
TraesCS6B02G414000.1	82.68	6.17	757
TraesCS6A02G377500.1	82.83	6.13	756
TraesCS6D02G362100.1	82.62	6.34	756
TraesCS6A02G377600.1	83.59	5.84	761
TraesCS6B02G415100.1	83.88	5.77	763
TraesCS6D02G362200.1	83.62	6.5	761

TraesCS5B02G521800.1	83.95	5.94	761
TraesCS5D02G520600.1	83.85	6.31	761
TraesCS1A02G012000.1	82.89	6.75	743
TraesCS1D02G010100.1	83.05	6.11	744
TraesCS1A02G011900.1	83.31	6.88	746
TraesCS1B02G050100.1	75.05	7.7	683
TraesCS6B02G010600.1	74.86	7.47	683
TraesCS2A02G071900.1	78.94	7.79	720
TraesCS2B02G087300.1	78.93	7.32	719
TraesCS5B02G363100.1	81.64	5.66	738
TraesCS5D02G477400.1	78.27	6.79	705
TraesCS1A02G017700.1	75.44	6.95	681
TraesCS1B02G021800.1	72.73	6.61	661
TraesCS1D02G016200.1	74.93	5.59	683
TraesCS1B02G075700.1	74.81	6.62	684
TraesCS1A02G058200.1	73.83	6.58	681
TraesCS1D02G058800.1	74.11	6.49	683
TraesCS2A02G093100.1	74.23	6.29	675
TraesCS4D02G042300.1	78.10	6.87	709
TraesCS4D02G042400.1	77.96	6.62	709
TraesCS6D02G001400.1	77.75	6.62	705
TraesCS6B02G009100.1	69.53	6.54	627
TraesCS2B02G563800.1	77.61	8.55	702
TraesCS7A02G565200.1	81.19	6.85	732
TraesCS7D02G545900.1	81.25	6.24	732
TraesCS2D02G002600.1	79.70	6.37	714
TraesCS3A02G533100.1	79.84	7.05	716
TraesCS3B02G595500.1	80.06	6.71	720
TraesCS7A02G062000.1	80.77	6.01	741
TraesCS6D02G395400.1	82.65	6.1	750
TraesCS6D02G395900.1	82.33	6.25	741
TraesCS3B02G098900.1	83.98	6.79	752
TraesCS2B02G563900.1	79.45	6.76	724
TraesCS6A02G061200.1	79.59	6.54	733
TraesCS6D02G063400.1	79.18	6.21	728
TraesCS5B02G458300.1	72.91	7.27	676
TraesCS5A02G445700.1	77.86	5.75	719
TraesCS5B02G454100.1	78.60	6.3	723
TraesCS5B02G452300.1	77.89	5.59	718
TraesCS5B02G454700.1	77.97	5.74	718
TraesCS2A02G381400.1	82.23	6.2	742
TraesCS6B02G100300.1	81.93	5.94	739

TraesCS6A02G225400.1	82.2	5.84	757
TraesCS6B02G256200.1	71.19	8.66	653
TraesCS6D02G210200.1	82.32	6.16	755
TraesCS6B02G413900.1	85.22	6.45	765
TraesCS2A02G047600.1	81.1	7.25	753
TraesCS4A02G351700.1	83.81	6.13	761
TraesCS2D02G070600.1	77.61	7.53	712
TraesCS2A02G071800.1	77.9	7	716
TraesCS1A02G016300.1	75.21	5.3	683
TraesCS1D02G014900.1	75.07	5.91	683
TraesCS1B02G020200.1	75.17	5.91	683
TraesCS1D02G016800.1	75.51	6.34	687
TraesCS6B02G015800.1	75.65	7.03	686
TraesCS5A02G464700.1	78.5	7.08	711
TraesCS5A02G052900.1	76.71	7.93	688
TraesCS5B02G063600.1	76.47	7.21	690
TraesCS2B02G231700.1	80.24	6.55	725
TraesCS6B02G055400.1	77.09	6.83	705
TraesCS2B02G151900.1	81.68	7.01	744
TraesCS5B02G455500.1	78.06	6.74	718

Table S2. The pairwise protein sequence comparison between the TaWAK-5D600 and other desease resistant WAK kinase proteins in crop plants.

Gene name	Gene ID	Disease name	Identity with TaRLK-5D600
ZmWAK-RLK1 (Htn1)	AKP45154.1	northern corn leaf blight	20%
ZmWAK-qHSR1	AIY68255.1	maize smut	27.05%
OsWAK-Xa4	KU761313.1	rice blast	28.25%
OsWAK1	Os01t0136400_01	rice blast	24.50%
OsWAK91	Os09t0561600_01	rice blast	31.06%
OsWAK92	Os09t0562600_01	rice blast	33.37%
OsWAK14	Os02t0632800_01	rice blast	14.49%
TaWAKL4-Stb6	TraesCS3A02G049500.2	Septoria tritici blotch	20.21%
TaWAK2A-800	TraesCS2A02G071800.1	Sharp eyespot and Fusarium head blight	33.33%
TaWAK6	TraesCS5B02G063600.1	leaf rust	32.63%
TaWAK-6D	TraesCS6D02G210200.1	Sharp eyespot and Fusarium crown rot	49.68%
TaWAK-7D	TraesCS7D02G087000.1	Sharp eyespot	40.65%

Table S3. Primers used in this study.

Primer name	Sequence	Useage
VIGS-TaWAK5DF	5'-TACGCTAGCCATCCCGTTCCCGTACGG-3'	Primer for BSMV-VIGS
VIGS-TaWAK5DR	5'-GACGCTAGCCCAGTGGCTGGGTGATAG3'	
BSMV-CPF	5'-TGACTGCTAAGGGTGGAGGA-3'	Primer for CP gene
BSMV-CPR	5'-CGGTTGAACATCACGAAGAGT-3'	
TaActin-RTF	5'-CGTCCATGATCCCTCCGAATT-3'	Primer for RT-qPCR
TaActin-RTR	5'-CTCCATGTCATCCCAGTTG-3'	
TaWAK-5DRTF	5'-CCTTTCAGTGATTTGGGCGC-3'	
TaWAK-5DRTR	5'-CGGTGGTGCAGTTGAGGTTA-3'	
TaSERK1-RTF	5'-TGGCAGTGCATAGGAACCTG-3'	
TaSERK1-RTR	5'-CCCAGTGCAATCCGAGTTCT-3'	
TaMPK3-RTF	5'-TACATGAGGCACCTGCCGCAGT-3'	
TaMPK3-RTR	5'-GGTTCAACTCCAGGGCTTCGTTG-3'	
TaChitinase3-RTF	5'-CCCACCCTAACCTGAGCATC-3'	
TaChitinase3-RTR	5'-ACTGGTTGATCATGGCGGAG-3'	
TaChitinase4-RTF	5'-GAAGTCCCCCATGGCGATC-3'	
TaChitinase4-RTR	5'-GGTCCCGCAATAACCGTACT-3'	
TaPR1-RTF	5'-AAACAGCAGCAACCCAAGAA-3'	
TaPR1-RTR	5'-GGGTCCAGTAGCACCGATTTA-3'	
GFP-TaWAK-5D	5'- GCCGGGGCAGCGGAATTCATGACGAAGCTGAGGTATTC-3'	subcellular localization
GFP-TaWAK-5D	5'- GATTTCAGCGTACCGAATTCTCAACGCCCTGATTGCAGATTTG-3'	subcellular localization
